# Vinorelbine-induced regression of a choroidal metastasis from primary breast carcinoma

**DOI:** 10.1186/s40942-018-0121-3

**Published:** 2018-05-14

**Authors:** Malvika Arya, Jay S. Duker

**Affiliations:** New England Eye Center, Tufts Medical Center, 800 Washington Street, Box 450, Boston, MA 02116 USA

**Keywords:** Choroidal neoplasm, Uveal neoplasm, Breast cancer, Vinorelbine, Antineoplastic agents phytogenic, Chemotherapeutic anticancer agents, Optical coherence tomography, OCT, Choroid

## Abstract

**Background:**

Various therapeutic options exist to treat choroidal metastatic lesions. However, they are all associated with potential long-term adverse effects. This case report discusses a case of choroidal metastasis from primary breast carcinoma that regressed after single-agent chemotherapy.

**Case presentation:**

We report a case of choroidal metastasis from estrogen receptor (ER) positive breast carcinoma that became resistant to endocrine therapy. The primary malignancy was treated with surgical resection and adjuvant chemoradiation, followed by hormone therapy with various agents in combination with kinase inhibitors for ER resistance. The choroidal metastatic lesion regressed after the initiation of vinorelbine. Vinorelbine is a cytotoxic vinca alkaloid with tolerable systemic adverse effects.

**Conclusions:**

This case report highlights the possible role of vinorelbine as a single chemotherapeutic agent for the conservative therapy of uveal metastasis from advanced breast carcinoma, irrespective of responsiveness to hormone therapy.

## Background

The choroid is the primary ocular site for metastatic cancer due to its rich vascular supply and fenestrated choriocapillaris [1–3]. In women, the breast is the predominant site of primary neoplasms, and choroidal metastatic lesions appear in approximately 8% of patients with breast carcinoma [3]. Choroidal metastatic lesions secondary to breast cancer are often bilateral and located close to the posterior pole [1]. Uveal metastatic lesions may appear during systemic dissemination and are associated with a limited life expectancy [1, 4].

60–70% of breast carcinomas are estrogen receptor (ER) positive and are responsive to endocrine therapy [5–7]. These tumors are treated with Tamoxifen in pre-menopausal women and aromatase inhibitors in post-menopausal women, often after surgical resection of the primary lesion. However, a persistent risk of tumor recurrence remains, either from loss of ER expression or from resistance to hormone therapy by a mutation in the ER pathway [8]. This study reports a case who developed a choroidal metastatic lesion, while on therapy with selective estrogen receptor modulators (SERMs) for ER positive breast carcinoma, which then regressed following systemic chemotherapy with vinorelbine.

## Case presentation

**Fig. 1:**
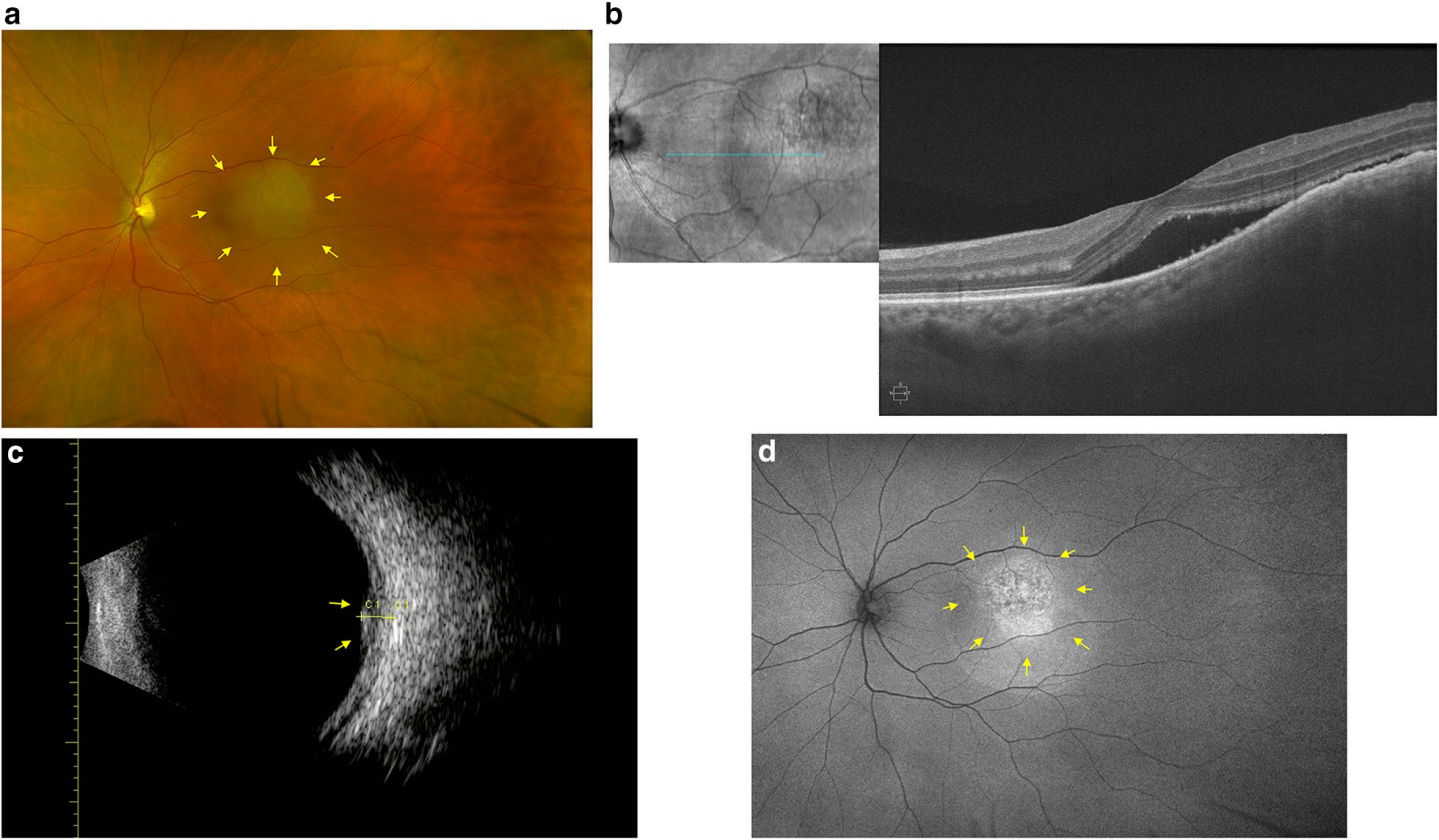
Imaging studies performed in June 2017 for a 58-year-old female with choroidal metastasis from primary breast carcinoma. **a** The extent of the yellow-colored choroidal mass superior and temporal to the macula is marked (yellow arrows). **b** Structural OCT demonstrated subretinal fluid associated with the choroidal mass. **c** Ultrasound showed a 2.47 mm choroidal lesion (yellow arrows). **d** Fundus autofluorescence demonstrated a hyper-fluorescent lesion (yellow arrows) with surrounding subretinal fluid

A 58-year-old female presented to New England Eye Center in June 2017 with decreased vision in her left eye of approximately 2 weeks duration. Her past ophthalmic history was significant for a retrobulbar migraine in her left eye. On presentation, her best-corrected visual acuity was 20/20 in the right eye, which stayed consistent throughout her follow-up visits, and 20/40 in the left eye. Funduscopic exam of the affected eye revealed a 5.8 mm in diameter, yellow-colored choroidal mass located superior and temporal to the macula, as shown in Fig. 1a. Optical coherence tomography (OCT) and ultrasound of the corresponding site revealed subretinal fluid associated with a 2.47 mm choroidal lesion with medium internal reflectivity (Fig. 1b, c). Fundus autofluorescence of the lesion also revealed a hyper-autofluorescent choroidal mass with a surrounding pocket of subretinal fluid (Fig. 1d). Imaging of the right eye was within normal limits.

Her medical history was significant for stage IIIA T3 N1 M0, ER positive, progesterone receptor (PR) positive, human epidermal growth factor receptor 2 (HER2) negative, well-differentiated invasive ductal carcinoma of the right breast. A tumor measuring 6 cm was first diagnosed by screening mammogram 16 years prior to ocular presentation, in May 2001. She subsequently underwent a modified radical mastectomy of the right breast with sentinel and axillary lymph node dissection in June 2001. Surgical margins were free of the tumor. One sentinel lymph node and three additional lymph nodes, with a total of 4 out of 12 lymph nodes, were positive for metastases. One lymph node showed extra-nodal extension. Consequently, localized radiation to the chest wall and supraclavicular region was completed, followed by 6 cycles of adjuvant CAF (cyclophosphamide, doxorubicin, 5-flourouracil) chemotherapy. She was treated with Tamoxifen 10 mg twice daily for 5 years following the completion of adjuvant chemoradiotherapy.

In January 2012, a surveillance CT scan of the chest revealed a 2.0 cm right upper lobe mass with hilar and mediastinal lymphadenopathy. Biopsy during mediastinoscopy confirmed metastatic adenocarcinoma consistent with breast carcinoma as the primary site, and displayed ER+, PR+, HER2− expressivity. Accordingly, the patient then completed nearly 3 years of anti-estrogen therapy with the aromatase inhibitor letrozole 2.5 mg daily.

Letrozole was discontinued due to recurrence of the disease in the right knee. As treatment for this metastasis, the patient underwent radical resection of the right distal femur and reconstruction with a prosthesis. The tumor was diffusely ER+, and the patient was started on anti-estrogen therapy with another aromatase inhibitor, exemestane 25 mg daily. 10 months later it was discontinued due to disease progression when a core biopsy of one of the inguinal lymph nodes revealed metastatic breast adenocarcinoma with ER 90%, PR 0%, and HER2 2+. The patient was then started on anti-estrogen therapy with the aromatase inhibitor fulvestrant in conjunction with the cyclin-dependent kinase inhibitor palbociclib for suspected resistance to ER endocrine therapy.

In early 2017, new lytic vertebral and left pelvic metastatic lesions were detected. The patient subsequently underwent radiation therapy to the T11-L1 region, left hip, and adjacent pelvis. She was restarted on exemestane, with the mTOR kinase inhibitor everolimus. This therapy was discontinued after 2–3 weeks due to profound weakness, nausea, and emesis. Bone scan showed a progression of osseous dissemination.

By June 2017, she had extensive osseous and lymph node dissemination. She was restarted on fulvestrant in combination with the receptor activator of nuclear factor kappa-B (RANK) ligand and osteoclast inhibitor denosumab. It was at this time that she was diagnosed with the left choroidal mass and referred for an ophthalmology consult. The patient was advised by oncology to discontinue fulvestrant due to disease progression, and was started on the mitotic inhibitor vinorelbine 25 mg/m^2^ IV weekly for 2–3 weeks as tolerated.

Regarding the newly diagnosed choroidal mass, after careful consideration of radiation therapy for more definitive treatment versus photodynamic therapy (PDT) or laser to treat the subretinal fluid, a conservative approach was adopted with a 1-month follow-up to assess for possible regression of the lesion in conjunction with the initiation of vinorelbine. In July 2017, visual acuity of the left eye improved to 20/30 and funduscopic exam and color fundus photo revealed a slightly regressed choroidal mass, at 4.75 mm, with a reduction in subretinal fluid. The OCT scan showed decreased subretinal fluid with the choroidal mass size stable at 2.47 mm. With a possible future consideration of external beam radiotherapy (EBR) or photodynamic therapy, the patient was closely monitored for an increase in choroidal mass size and surrounding exudate in the event of a waning effect of vinorelbine.

**Fig. 2:**
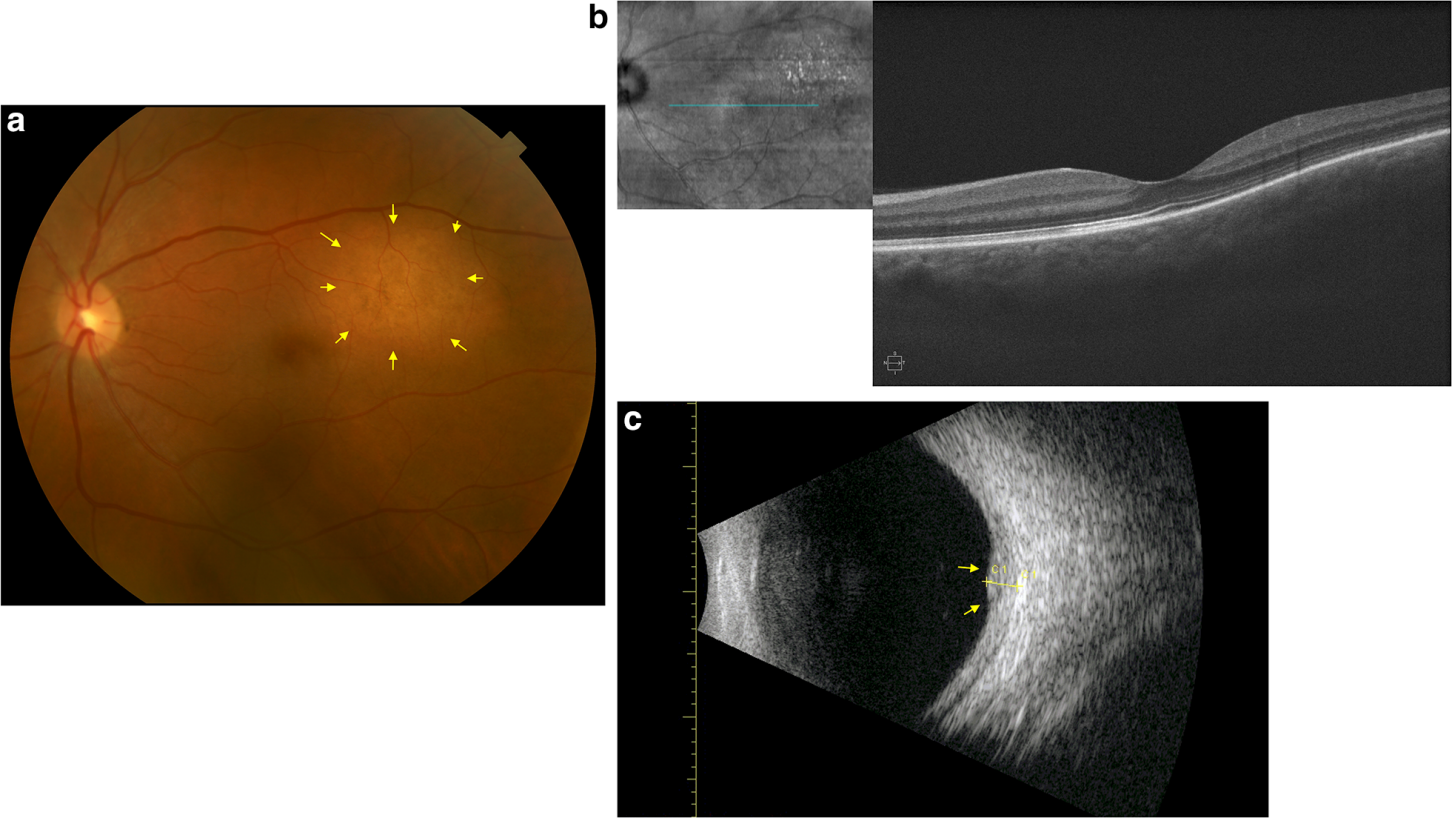
Imaging studies performed in September 2017 for the 58-year-old female with choroidal metastasis from primary breast carcinoma. **A** The yellow-colored mass (yellow arrows) located superior and temporal to the macula appeared to have slightly regressed. **B** Subretinal fluid associated with the choroidal lesion in June 2017 appeared to have resolved. **C** Ultrasound demonstrated a stable size of the choroidal mass at 2.47 mm (yellow arrows)

During her last follow-up in September 2017, visual acuity of the left eye improved to 20/20. Funduscopic exam and color fundus photo revealed a regressed yellow-colored, temporally located choroidal mass (Fig. 2a). OCT demonstrated resolution of the subretinal fluid with a stable mass size of 2.47 mm seen on ultrasound (Fig. 2b, c). The patient continued treatment with vinorelbine and denosumab for ER+, PR−, stage IV metastatic breast carcinoma and had completed 6 cycles of vinorelbine. A conservative approach was continued with a plan for frequent follow-up.

## Discussion and conclusions

Choroidal metastatic lesions in primary breast carcinoma usually occur with systemic dissemination of the malignancy to other organs. In some patients, uveal metastatic lesions may remain asymptomatic and thus, undiscovered or discovered as a scar lesion after systemic therapy with different chemotherapeutic agents. In such cases, the systemic treatment may control the ocular disease before the ocular diagnosis.

Treatment of choroidal metastatic lesions is guided by the presence of visual symptoms. Possible treatment options for uveal metastases include systemic chemotherapy, immunotherapy, whole eye or localized radiation, indocyanine green (ICG) augmented transpupillary thermotherapy (TTT), anti-VEGF injections, and/or surgical resection. Due to lack of controlled clinical trials, there is no gold standard for their management, and therapy is driven by the treating physician’s clinical judgment. Studies have demonstrated that uveal metastatic lesions may be responsive to systemic chemotherapy [9–12], with regression of subretinal fluid visualized on OCT as well [13, 14], decreasing the need for local radiation therapy [11]. Therefore, asymptomatic uveal metastatic lesions may be followed conservatively, while the patient is being treated with systemic chemotherapy.

The therapy and prognosis of primary breast carcinoma are guided by its clinicopathological characteristics, the presence or absence of hormone receptors, the expression of the HER2 oncogene, and multiparameter gene expression assays. Chemotherapy following the resection of primary breast carcinoma is recommended in all patients with high risk characteristics, such as a large tumor burden, positive lymph nodes, and/or HER2 negative status with the absence of hormone receptors. 75% of breast carcinomas are ER and PR positive [15], and these tumors demonstrate a good response to endocrine therapy. Hormone therapy with Tamoxifen in pre-menopausal women up to 5 years has been shown to reduce the risk of recurrence and decrease mortality [16, 17]. Tamoxifen and fulvestrant are SERMs, namely estrogen antagonists that downregulate the ER protein in malignant cells. Tamoxifen is preferred in premenopausal women to preserve fertility, but comes with a risk of uterine malignancy and thromboembolism. In post-menopausal women, aromatase inhibitors are preferred to reduce the risk of recurrence, with better tolerability and without the life-threatening adverse effects of partial estrogen agonists, such as Tamoxifen [18]. Aromatase inhibitors decrease estrogen production by blocking the peripheral conversion of adrenal androgens. Tamoxifen therapy is usually recommended for 5 years; however, extended therapy for 10 years has demonstrated an additional 3% improvement in breast cancer mortality [19]. Patients with high risk disease are usually recommended an anthracycline and taxane containing regimen, whereas those patients with low to moderate risk may be treated with a taxane (paclitaxel and docetaxel) regimen only [20]. Patients with HER2 positive disease are recommended trastuzumab and pertuzumab monoclonal antibodies in addition to chemotherapy [21].

Patients with ER+, PR+ and HER2− malignancy usually receive multiple cycles of endocrine therapy before transitioning to a single chemotherapeutic agent. Our patient developed a choroidal metastasis in ER+, PR+ and HER2− breast carcinoma while on hormone therapy with fulvestrant. EBR therapy has a response rate of 63–83% for tumor regression [22], and was considered in this patient. However, EBR may be associated with the long-term risks of exposure keratopathy, iris neovascularization, radiation retinopathy, and optic neuropathy [23]. Arguably, the goal of therapy in metastatic disease, including uveal metastases, is mainly palliative, aiming to extend life while minimizing the adverse effects of chemotherapy and other therapeutic interventions. Therefore, in our patient, a conservative approach was adopted, accompanied by the introduction of vinorelbine, a single chemotherapeutic agent with good tolerability. With this approach, the patient has demonstrated slow, but continued resolution of the uveal metastatic lesion. To the best of our knowledge, this is the first case to report regression of choroidal metastasis with vinorelbine in an ER+, but hormone therapy resistant primary malignancy.

ER+ metastases, such as choroidal tumors, are usually responsive to Tamoxifen or aromatase inhibitor therapy. However, some ER+ tumors are either only partially responsive to SERMs, due to ER-expression paucity, or become resistant to endocrine therapy, due to a possible mutation in kinase enzymes in the ER expression pathway, as seen in our patient. Wong et al. [11] reported complete regression of a choroidal metastasis from ER−, HER2+ breast carcinoma treated with trastuzumab and vinorelbine. Vinorelbine is a vinca alkaloid that is a semisynthetic derivative of vinblastine, with less neurotoxicity than vinblastine. Vinorelbine inhibits DNA replication mainly by preventing the assembly of microtubules in malignant cells and disrupting cell membrane function [24]. It may be a form of single-agent chemotherapy that is reasonably well-tolerated and administered weekly, with a risk of possible mild neurological, hematological, and gastrointestinal toxicity.

The regression of metastatic lesions, either partial or complete, during systemic therapy directed towards the primary malignancy is a well-known occurrence. However, the spontaneous regression of tumors without therapy has also been seen, although infrequent. In a series of 254 patients with choroidal metastasis from breast cancer, 18% of the patients were followed conservatively without intervention. The metastatic lesion regressed in 42% of these patients, while remaining stable in 33% of this cohort [25]. The natural regression of tumors may be attributed to programmed cell death by apoptosis, an increased presence of natural killer cells in circulation, or a modification of the tumor microenvironment that prevents tumor proliferation by inhibiting matrix metallo-proteinases and angiogenesis [26]. Additionally, p. 53 oncogene-mediated apoptosis is a pathway known to be more active in estrogen-dependent breast carcinomas [27]. Thus, spontaneous regression may have been a possible contributing factor in this case. However, since regression coincided with the initiation of vinorelbine, it was the most likely contributor to the observed regression.

Overall, we concur with earlier reports of a conservative approach for choroidal metastatic lesions in disseminated breast carcinoma while on chemotherapy [9–11, 28, 29], Additionally, we suggest the consideration of vinorelbine therapy in patients with choroidal metastasis, irrespective of hormone receptor status and responsiveness to antiestrogen therapy in disseminated breast malignancy, thereby limiting local radiation therapy to vision threatening lesions only.

## Abbreviations

ER: Estrogen receptor
SERM: Selective estrogen receptor modulator
OCT: Optical coherence tomography
HER2: Human epidermal growth factor receptor 2
PDT: Photodynamic therapy
ICG: Indocyanine green
TTT: Transpupillary thermotherapy
